# The London Hospital

**Published:** 1916-03-04

**Authors:** 


### THE LONDON HOSPITAL.

### Some Statistics of the Year 1915.

The in-patients in 1915 were 18,933, 600 up, and the out-patients were 149,507, about 20,000 down. Over 19,000 anæsthetics were given in 1915, and only two deaths occurred.

### St. Anthony Wards.

Before the war the Grocers' Company, to whom this hospital already owes the Grocers' Wing, had generously presented the hospital with wards for the Salvarsan treatment of syphilis. It has not been able to open them because of the war. But Dr. Sequeira, the member of our staff who is at the head of that department, has drawn the Committee's attention to the very serious increase of syphilis, which to-day is endangering the welfare and future of the British people. Seeing that this must be stopped, it has been decided to open these wards at once; and, providing ample funds are forthcoming, they will be worked to their fullest extent. Unfortunately the last year's accounts show that the London Hospital has spent £14,000 more than it received in 1915. The increased cost of provisions accounts for nearly £10,000 of this.

### Some Welcome Visitors.

The distinguished Russian journalists and authors who are visiting this country as guests of the Government paid an extensive visit to and went all over the hospital the other day. Amongst them were M. Nemirovitch, M. Yegoroff, M. Bashmakoff, M. Chukovsky, Count Alexis Tolstoy, and M. Vladimir Nabokoff. They were accompanied by Sir Thomas Barlow, Sir Rickman Godlee, President of the College of Surgeons, Sir Ronald Ross, Sir David Bruce, and several other leaders of the profession in England. The Japanese Nursing Unit, who have been working at the expense of the Japanese Red Cross at Netley Hospital, went to the London Hospital, and were very pleased with their visit.

### Increased Cost of Drugs.

It is interesting to note that the price of drugs has increased to an absolutely abnormal extent, for instance: Cod-liver oil—price was 71s. a barrel, now 350s. a barrel; atropine sulphate was 10s. 6d. an oz., now 140s. an oz.; aspirin was 1s. 10½d. a lb., now 48s. a lb.; carbolic acid was 3¾d. a lb.; now 1s. 4½d. a lb.; salicylic acid was 1s. a lb., now 20s. a lb.; 1-pint medicine bottles were 15s. a gross, now 36s.; test-tubes were 2s. 2d. a gross, now 6s. 6d. These are merely a few examples, which might be multiplied indefinitely.

### The Financial Position.

The meat bill in 1915 was nearly £3,000 up, milk nearly £2,000, coal nearly £2,000, grocery nearly £1,000, butter and cheese £1,300, fish and poultry £700, and vegetables nearly £400. In addition to these expenses the

London had to pay an additional £2,500 in medical salaries. On the other hand, the cleaning bill has been cut down by £2,000, and dressings by £650, owing to extreme care exercised in the wards. The legacies have dropped from £27,291 in 1914 to £8,859 in 1915. The total income dropped from £133,000 in 1914 to £124,000 in 1915; whilst the expenditure was £139,000 in 1915, as against £124,000 in 1914.

Nurses and Nursing Statistics.

On December 31, 1915, the complete total of the nursing staff numbered 799, of which 340 were probationers, so the committee congratulate the Board on having been able to send 146 to help nurse our soldiers, although it has been necessary to increase the beds and to devote 250 beds to soldiers and 250 to sailors. Of the nurses employed, no less than 105 are daily away from duty on holidays, on days off, or on sick leave. The nurses have three hours off every day instead of the usual two. Ten of the London nurses have been mentioned in despatches since the last quarterly court, and three of them were killed by the accident to H.M. ship *Natal.* The Edith Cavell Fund of the "Daily Mirror" now approximates £10,000. Two hundred and seventy young women have been trained for three months, and have largely been absorbed by the War Office as probationers in military hospitals, and have done most excellent work. The increase in the nurses has involved further capital expenditure on new nursing kitchens.

